# Effect of Non-Rotavirus Enteric Infections on Vaccine Efficacy in a ROTASIIL Clinical Trial

**DOI:** 10.4269/ajtmh.23-0348

**Published:** 2024-04-16

**Authors:** Dilip Abraham, Prasanna Samuel Premkumar, James A. Platts-Mills, Tushar Tewari, Niranjan Bhat, Revathi Rajendiran, Hemavathi Gunalan, Gagandeep Kang

**Affiliations:** ^1^ The Wellcome Trust Research Laboratory, Division of Gastrointestinal Sciences, Christian Medical College, Vellore, India;; ^2^ Infectious Diseases and International Health, University of Virginia, Charlottsville, Virginia;; ^3^ Center for Vaccine Innovation and Access, PATH, New Delhi, India;; ^4^ Center for Innovation and Access, PATH, Washington, District of Columbia

## Abstract

This study examined the relative proportion of enteric pathogens associated with severe gastroenteritis (GE) among children younger than 2 years in a phase III efficacy trial of the ROTASIIL^®^ vaccine in India, evaluated the impact of co-infections on vaccine efficacy (VE), and characterized the association between specific pathogens and the clinical profile of severe GE. Stored stool samples collected from cases of severe GE in the phase III trial were tested by quantitative polymerase chain reaction using TaqMan^™^ Array Cards. Etiology was attributed by calculating the adjusted attributable fraction (AF) for each pathogen. A test-negative design was used to estimate VE. The pathogens with the highest AFs for severe diarrhea were rotavirus (23.5%), adenovirus 40/41 (17.0%), *Shigella* spp./enteroinvasive *Escherichia coli*, norovirus GII, enterotoxigenic *E. coli,* and *Cryptosporidium* spp. A considerable proportion of the disease in these children could not be explained by the pathogens tested. Severe GE cases associated with rotavirus and *Shigella* spp. were more likely to have a longer duration of vomiting and diarrhea, respectively. Cases attributed to *Cryptosporidium* spp. were more severe and required hospitalization. In the intention-to-treat population, VE was estimated to be 43.9% before and 46.5% after adjustment for co-infections; in the per-protocol population, VE was 46.7% before and 49.1% after adjustments. Rotavirus continued to be the leading cause of severe GE in this age group. The adjusted VE estimates obtained did not support co-infections as a major cause of lower vaccine performance in low- and middle-income countries.

## INTRODUCTION

The global introduction of rotavirus vaccines and their widespread use starting in 2006 have ameliorated the disease burden due to rotavirus diarrhea to a great extent, as demonstrated in multiple impact assessment studies and vaccine effectiveness trials in communities around the world.^1^^–^^4^ However, rotavirus remains a significant cause of diarrhea despite substantial reductions in the burden of rotavirus gastroenteritis (RVGE) both globally^1^^,^^5^^,^^6^ and in India.^3^^,^^7^ For example, an effectiveness study carried out in Tanzania observed that rotavirus remained the leading cause of diarrhea requiring hospitalization even 2 years after introduction of the rotavirus vaccine in a national immunization program.^2^

Oral rotavirus vaccines, when tested in low- and middle-income countries (LMICs), are consistently found to have lower efficacy compared with trials using the same products in high-income countries. A recent meta-analysis of ROTARIX^®^ and RotaTeq^®^ vaccine efficacy (VE) trials categorized by the under-5 mortality rates of the country where the trial was held, showed a distinct demarcation, with lower VEs reported in higher mortality settings.^8^ In India, phase III clinical trials of ROTAVAC^9^ and ROTASIIL^10^ vaccines demonstrated VEs against severe RVGE (SRVGE) of 53.6% (95% confidence intervals [CI] - 35.0%–66.9%) and 39.5% (26.7%–50.0%), respectively.

Multiple factors have been proposed to account for poor VE in LMICs, including the presence of interfering maternal antibodies from breast milk, impaired immunity owing to nutritional deficiencies, environmental enteric dysfunction, genetic factors, and co-infections.^11^^,^^12^ The presence of co-infecting pathogens may interfere with the uptake and replication of live oral vaccines or may have immunomodulatory effects that can negatively affect immunogenicity and efficacy.^13^^,^^14^ Breakthrough severe gastroenteritis (GE) cases in the trial may have been misclassified as rotavirus cases owing to the presence of rotavirus in the samples but were in actuality caused by pathogens other than rotavirus; this could have led to an underestimation of VE.

Quantitative output from the TaqMan Array Card (TAC) assay (Thermo Fisher Scientific, Carlsbad, CA) has enabled the development of models that have been instrumental in understanding the etiology of disease from samples that could contain multiple pathogens and commensals, even in the absence of disease. This could help us to understand the role of co-infections in the attenuation of rotavirus VE in LMICs, as well as to determine the overall distribution of other significant agents causing severe diarrhea in children from this age group across India.^15^ The ROTASIIL efficacy trial in India^10^ provides an opportunity to examine these questions using this methodology.

The parent BRV-PV (ROTASIIL; Serum Institute of India Pvt Limited, Pune, India) phase III efficacy study was initiated in 2014 and does not reflect the effect of the introduction of the rotavirus vaccine in India’s Universal Immunization Program in 2016.

## MATERIALS AND METHODS

### Study population and sample selection.

The BRV-PV phase III trial aimed to measure the clinical efficacy of ROTASIIL to prevent SRVGE in children younger than 2 years (ClinicalTrials.gov NCT 02133690; Clinical Trial Registry of India [CTRI/2013/05/003667]). Details of this trial and the results obtained have been described.^10^ Briefly, 7,500 infants were randomized to receive either three doses of BRV-PV vaccine or placebo at 6, 10, and 14 weeks of age and were subsequently followed up until the development of SRVGE, which was the primary endpoint for the trial; these children were later followed up until 2 years of age. Gastroenteritis was defined as the occurrence of at least three watery or looser-than-normal stools within a 24-hour period with or without vomiting, and the Vesikari Scoring System was used to assess severity.^16^ Gastroenteritis cases with a Vesikari score ≥11 were classified as severe, and those with a score ≥15 were classified as very severe. Stool samples were tested for rotavirus by an ELISA assay. Aliquots of these stool samples were stored for further testing.

This analysis was carried out in data obtained from the trial after infants reached 2 years of age, which was the follow-up period in the efficacy study. This ancillary study was designed primarily to estimate the incidence and relative proportion of various etiological agents known to cause GE in the first 2 years of life and to attempt to correlate the severity of diarrhea with the pathogen identified in the analysis and the treatment arm. The endpoint for the original trial was SRVGE cases after the final vaccine dose, whereas in this reanalysis all SRVGE cases after the initial dose (intention-to-treat population [ITT]) were included along with all non–severe RVGE and severe non-RVGE cases. Three groups of samples were included in the study: 1) all rotavirus-positive samples from severe GE that represented the first SRVGE for the participant; 2) all rotavirus-positive samples from GE of any severity irrespective of the timing of sample collection; and 3) all samples from severe GE cases that tested negative for rotavirus.

### Sample storage and retrieval.

The sample aliquots were stored at The Wellcome Trust Laboratory at Christian Medical College, Vellore, in 2-mL vials at −80°C in freezers that were continually monitored for variations in temperature. At least 1 mL of stool (watery) or 0.5 g (semisolid) was required for inclusion in the study.

### Laboratory assays.

Total nucleic acid was extracted from the sample using the QIAamp Fast DNA Stool Mini Kit (Qiagen, Hilden, Germany) with a modified protocol that incorporated bead beating in the extraction process.^17^ Phocine herpesvirus and the MS2 bacteriophage were spiked into each sample prior to extraction as an internal control.

The TAC assay is an arrayed format of duplex quantitative polymerase chain reaction (qPCR) assays in a microfluidic card system, where separate wells contain pathogen or internal control–specific primers and probes (Supplemental Table 1). The TAC assays were carried out using the QuantStudio^™^ 12K Flex or a QuantStudio 7 Flex real-time PCR system. Testing for rotavirus was not included in the TAC as these data were already available from the original efficacy study. Each extraction batch included a negative control, which was tested in the TAC panel to exclude contaminants; if the control tested positive for any of the targets, the extraction was repeated for that batch and retested.

## STATISTICAL ANALYSES

To estimate GE disease etiology, the attributable fraction (AF) for each pathogen was estimated, adjusting for the effects of other pathogens that act as confounders in this analysis.^18^ Because samples from asymptomatic children were not collected in the efficacy trial, models derived from the qPCR reanalysis of the GEMS (Global Enteric Multicenter Study) case-control study^19^ were used to calculate pathogen-specific odds ratios (ORs), an approach that has been used in other studies with similar designs.^15^^,^^20^^,^^21^ The pathogen-specific ORs were calculated by fitting a multivariable conditional logistic regression model to describe the association between pathogen quantity and diarrhea, adjusting for the presence of other pathogens. Attributable fractions and their variance were calculated as described previously.^15^ The collection of samples from all GE cases in the ITT population over the period of 2 years enabled the estimation of attributable incidence (AI) for each pathogen, estimated as a measure of its contribution to disease etiology, by dividing the number of pathogen-specific episodes by the person-time observed.

To analyze the relationship between the clinical characteristics of the disease and the disease pathogen, rotavirus detection by antigen ELISA was modeled for presence/absence as a binary variable, whereas pathogens other than rotavirus were modeled as continuous AF values for each episode in a generalized linear mixed-effects model, scaled such that the OR for a one-unit change in AF corresponded to an episode attributable to that pathogen or pathogen category. To account for correlation among multiple episodes of GE in children, generalized estimating equations were used to fit logistic or ordinal logistic regression models.

A test-negative case-control design was used to recalculate the VE after adjusting for the effect of co-infections by various enteropathogens.^4^^,^^15^^,^^20^^,^^21^ Episodes of severe GE that were RV positive by enzyme immunoassay (EIA) were taken as cases, whereas those who tested negative in severe GE cases were designated as controls; vaccination status, study site, terms for age of the child, and quarter of the calendar year were included as predictors in a logistic regression model. An interaction term was added in between vaccination status and sum of the attributions to non-rotavirus enteropathogens. Vaccine efficacy was calculated as (1 − OR*i*) × 100, where OR*i* is the exponent of the model coefficient for vaccination status. Vaccine efficacy was calculated again as above but without including the interaction term, which was interpreted as the VE when the sum attribution to non-rotavirus enteropathogens was zero, followed by bootstrapping the data to calculate the difference in VE and variance estimates.

## RESULTS

### Details of samples selected.

**Figure 1.**
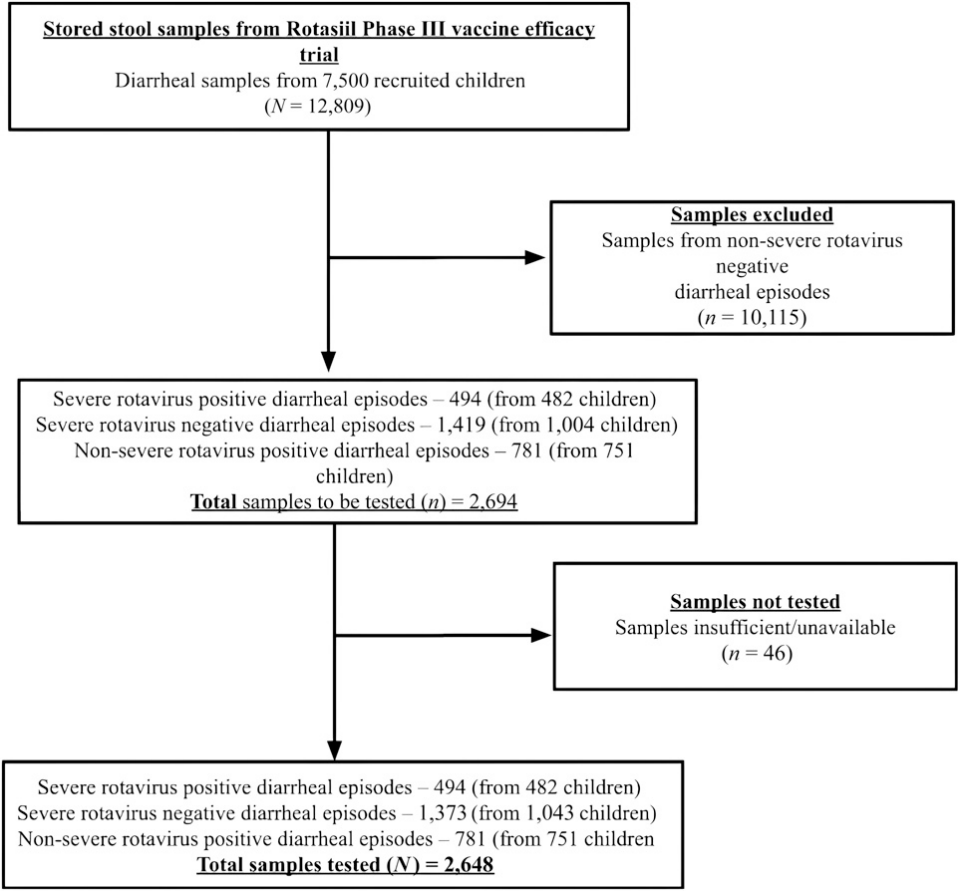
Selection of samples for testing from the parent ROTASIIL efficacy trial.

Per the criteria for inclusion into the three groups, 2,694 samples were selected to carry out reanalysis (Supplemental Table 2), of which 1,307 samples were from study participants in the vaccine group and 1,387 were from the placebo group. Samples that could not be tested because of unavailability were considered missing at random, and no imputation of values was made for these observations. In all, 2,648 samples were tested by the TAC assay (Figure 1). These consisted of 1,248 samples from the vaccinated group, of which 563 were RV-positive, and 1,364 from the placebo group, of which 712 were RV-positive.

The cases from which the samples were tested were not biased by sex, rotavirus positivity, or vaccination status (Supplemental Table 3). The age of these children at the time of GE ranged from 42 to 734 days, and the mean age for these cases was 364.3 days. Of these cases, 18.7% were SRVGE, with 15.8% having a Vesikari score of 15 or above, and 26.8% required hospitalization. About half of the cases also presented with 2–4 days of vomiting, and severe dehydration was seen in 10.2% of cases.

### Etiological fraction of enteropathogens for severe diarrhea.

The leading pathogens with the highest AFs for etiology of severe diarrhea were rotavirus (23.5%; 95% CI: 15.3%–25.4%), adenovirus (22.4; 95% CI: 17.2–27.1), *Shigella* spp./enteroinvasive *Escherichia coli* (EIEC), norovirus GII, stable toxin enterotoxigenic *E. coli* (ST-ETEC), and *Cryptosporidium* spp. The relative proportions of each enteropathogen contributing to GE are listed in Table 1.

**Table 1:** Etiology of severe GE (all cases) as determined by TAC qPCR in the context of rotavirus vaccination

Pathogen	AF	95% CI
Rotavirus	23.5	15.3–25.4
Adenovirus 40/41	17.0	13.1–20.5
*Shigella/*EIEC	7.9	6.0–12.0
Norovirus GII	3.9	0.0–8.4
ST/ETEC	3.8	2.3–4.9
*Cryptosporidium*	3.3	2.2–4.1
Sapovirus	2.2	0.0–8.9
Astrovirus	2.2	0.0–3.9
*C. jejuni/coli*	1.9	0.0–4.1
*V. cholerae*	0.6	0.3–0.7
*Aeromonas* spp.	0.6	0.0–2.5
tEPEC	0.3	0.1–0.5
*Salmonella* spp.	0.2	0.1–0.2
*E. histolytica*	0.04	0.02–0.05

AF = attributable fraction; *C. jejuni/coli = Campylobacter jejuni/coli*; *E. histolytica = Entamoeba histolytica*; EIEC = enteroinvasive *Escherichia coli*; GE = gastroenteritis; ST-ETEC = stable toxin enterotoxigenic *E. coli*; TAC = TaqMan Array Card; tEPEC = typical enteropathogenic *E. coli*; *V. cholerae = Vibrio cholerae.*

### Attributable incidence of severe GE.

**Figure 2.**
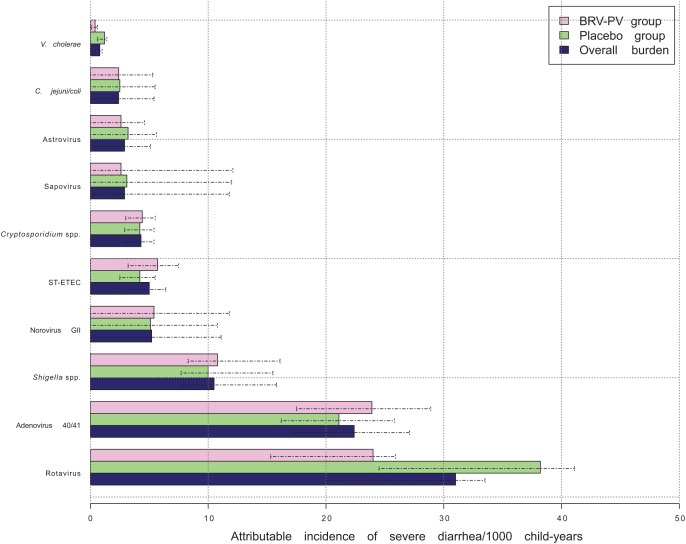
Attributable incidence (AI) per 1,000 child-years for severe gastroenteritis by study group and overall in children less than 24 months old. BRV-PV group: AI in participants who were administered the vaccine; Overall burden: overall AI of various pathogens for severe diarrhea; Placebo group: AI in participants who were administered placebo. Dashed lines indicate the 95% confidence intervals for each estimate. *C. jejuni/coli* = *Campylobacter jejuni/coli*; ST ETEC = stable toxin enterotoxigenic *Escherichia coli*; *V. cholerae* = *Vibrio cholerae*.

The most common pathogens contributing to the overall incidence of severe GE as measured by AI per 1,000 child-years in children ≤2 years were rotavirus (31.0 attributable episodes per 1,000 child-years; 95% CI: 20.1–33.5), adenovirus (22.4; 95% CI: 17.2–27.1), *Shigella* spp., norovirus GII, and ST-ETEC (Figure 2, Supplemental Table 4).

Among the various pathogens analyzed, viruses were the most common cause of GE with an AI of 64.4 (49.0% of the overall incidence), followed by bacteria with an AI of 18.7 (14.2%) and *Cryptosporidium* spp. with an AI of 4.3 (3.3%). However, when taken together, these pathogens contributed to only 66.4% of the overall incidence of severe GE, and approximately 33% of GEs were not attributed to any of the pathogens investigated.

Rotavirus was the principal etiology of severe GE, and the difference in AI between the vaccine (24.0; 95% CI: 15.3–25.9) and the placebo group (38.2; 95% CI: 24.5–41.1) was not statistically significant. In the vaccinated group, the burden of adenovirus 40/41 GE was similar to that of rotavirus in the vaccinated group (23.9; 95% CI: 17.5–28.9) (Figure 2).

### Attributable incidence of severe diarrhea by age

**Figure 3.**
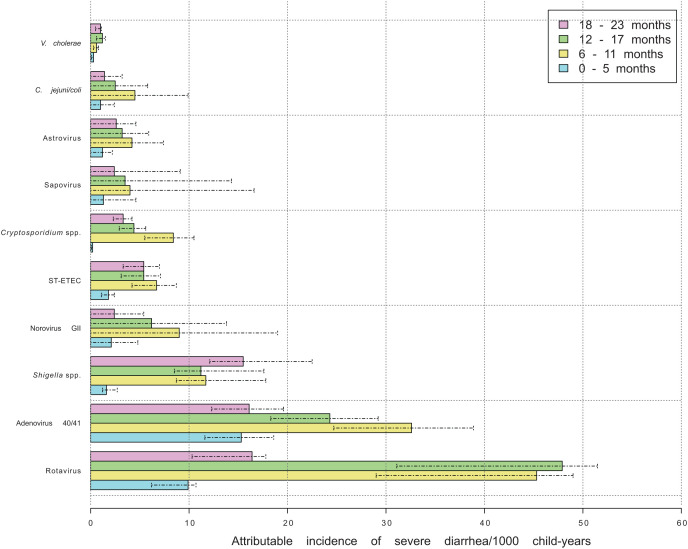
Attributable incidence per 1,000 child-years for severe gastroenteritis in children less than 24 months old stratified by age. Dashed lines indicate the 95% confidence intervals for each estimate. *C. jejuni/coli* = *Campylobacter jejuni/coli*; ST ETEC = stable toxin enterotoxigenic *Escherichia coli*; *V. cholerae* = *Vibrio cholerae*.

Rotavirus was accountable for the highest contribution to the pathogen-specific AI in most age groups (except for 0–5 months), followed by adenovirus 40/41 (Figure 3). *Shigella* spp. had a higher AI in children 18–23 months of age, where it was similar to the incidence of adenovirus 40/41 and rotavirus, and an inverse relationship was observed such that as age increased, the AI of rotavirus and adenovirus decreased.

### Associations between pathogen-specific AF and clinical profile of severe GE.

**Figure 4.**
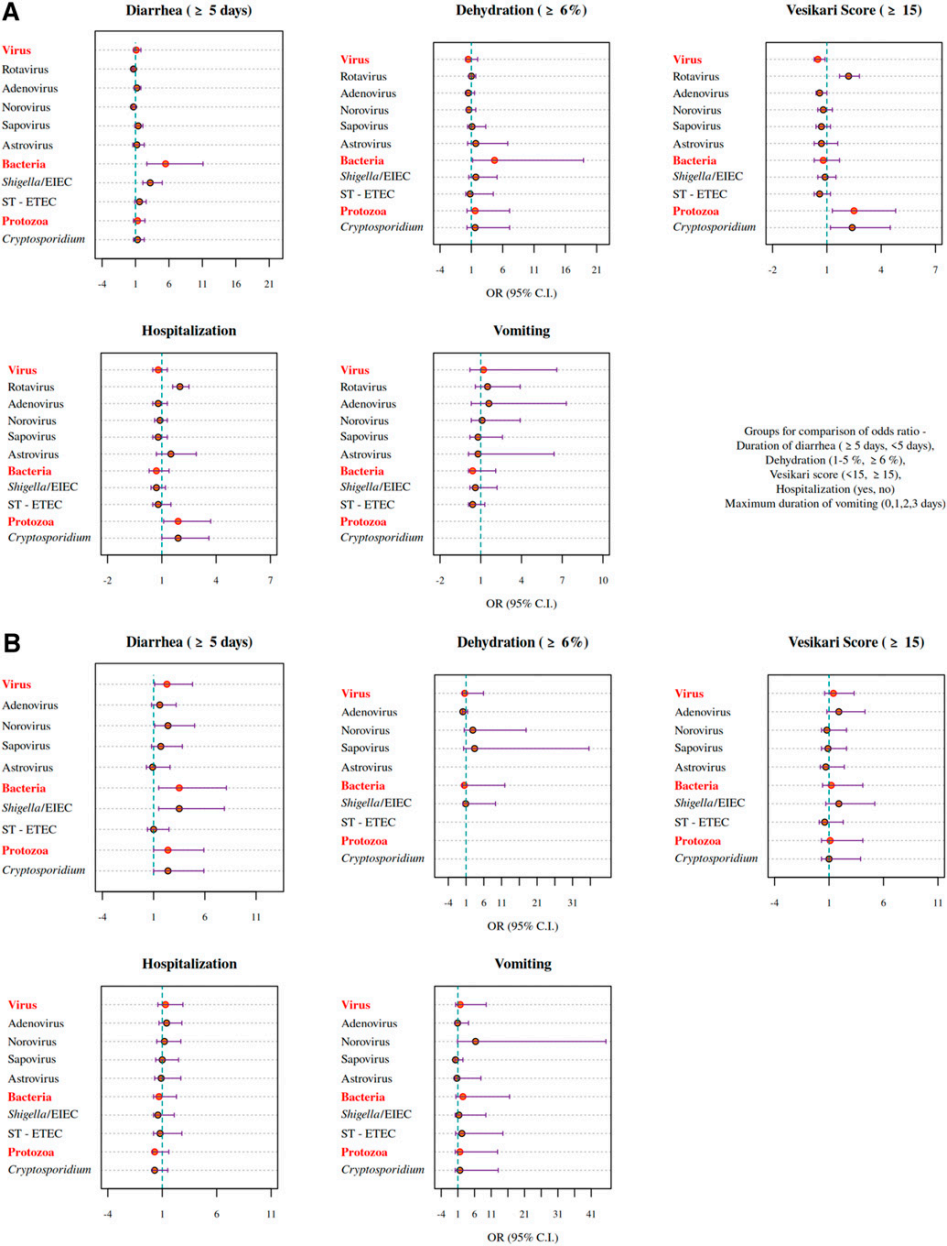
(**A**) Clinical profile of gastroenteritis attributable to specific pathogens. (**B**) Association between co-infecting pathogens and the clinical profile of rotavirus gastroenteritis. OR Virus = sum of AFs for adenovirus 40/41, norovirus GII, sapovirus, and astrovirus; Bacteria = sum of AFs for *Shigella*/EIEC, *Vibrio cholerae*, ST-ETEC, *Campylobacter jejuni/coli*, typical enteropathogenic *Escherichia coli*, Aeromonas, and *Salmonella*; Protozoa = sum of AFs for *Cryptosporidium* spp. and *Entamoeba histolytica.* AF = attributable fraction; EIEC = enteroinvasive *E. coli*; OR = odds ratio; ST-ETEC = stable toxin enterotoxigenic *E. coli.*

Severe rotavirus cases were associated with Vesikari scores of ≥15 (OR: 2.2; 95% CI: 1.7–2.8) compared with other pathogens. Severe GE cases attributed to *Shigella* spp. were more likely to develop diarrhea that persisted for more than 5 days (OR: 3.2; 95% CI: 2.0–5.0). *Cryptosporidium* spp. as an etiology of diarrhea displayed a profile of very severe GE cases that were more likely to receive a Vesikari score ≥15 (OR: 2.4; 95% CI: 1.2–4.5), and these patients were more likely to be hospitalized (OR: 1.9; 95% CI: 1.0–3.6) (Figure 4A). Overall, bacterial infections were associated with a longer duration of diarrhea (OR: 5.5; 95% CI: 2.7–11.1) and dehydration (OR: 4.7; 95% CI: 1.2–18.9), whereas protozoan infections were more often associated with severe diarrhea as measured by the Vesikari score (OR: 2.5; 95% CI: 1.3–4.8) and with hospitalization (OR: 1.9; 95% CI: 1.1–3.7).

Associations between the clinical profile of RVGE cases of any severity and co-infections as measured by their presence in stool samples in diarrhea-associated quantities were also estimated (Figure 4B). Diarrhea of longer duration was associated in RVGE with co-infections attributed to *Shigella*/EIEC (OR: 3.5; 95% CI: 1.5–7.9), norovirus (OR: 2.4; 95% CI: 1.1–5.0), and *Cryptosporidium* spp. (OR: 2.4; 95% CI: 1.0–5.9).

### Effect of co-infections on VE.

Vaccine efficacy was calculated from the ITT and per-protocol (PP) populations of study participants who developed severe rotavirus cases followed up until 2 years of age. There were 482 cases of severe or very severe rotavirus cases, of which 446 cases were categorized as falling in the PP group. The VE against SRVGE for the PP population was estimated to be 46.7% (95% CI: 32.7%–57.8%) with this analysis, compared with an efficacy of 39.5% (95% CI: 26.7%–50.0%) in the parent study. When adjusted for co-infections, the efficacy was calculated to be 49.1% (27.6%–64.2%) in PP participants. The difference with CI estimates is 2.4% (95% CI: −14.4% to 15.3%). Adjusted VE estimates were also calculated in the ITT population, and the results showed changes similar to those in the PP population (Table 2).

**Table 2:** Vaccine efficacy estimates for the ITT and PP populations

Measured Population	Adjusted VE	Adjusted VE with Co-Infection	VE Difference	VE in Parent Study (SRVGE)
ITT VE (95% CI)	43.9% (29.6% to 55.2%)	46.5% (24.6% to 62.0%)	2.6 (−14.4% to 15.4%)	38.8% (26.4% to 49.0%)
PP VE (95% CI)	46·7% (32.7% to 57.8%)	49.1% (27.6% to 64.2%)	2.4 (−14.4% to 15.3%)	39.5% (26.7% to 50.0%)

ITT = intention-to-treat; PP = per-protocol; SRVGE = severe rotavirus gastroenteritis; VE = vaccine efficacy.

## DISCUSSION

Rotavirus remained the leading cause of severe GE in this analysis among both vaccinated and placebo groups, comparable to results obtained from a corresponding reanalysis of specimens from an efficacy trial of ROTAVAC.^15^ A similar study in Tanzania examining the impact of the introduction of ROTARIX showed a reduction in diarrhea admissions; however, rotavirus remained the leading pathogen that caused diarrhea in children under 5 years requiring hospitalization.^2^ Geographical differences notwithstanding, the impact of rotavirus vaccine in reducing severe GE and preventing hospitalizations has been examined in multiple studies after the availability and introduction of these vaccines to national public health programs, and this effect of vaccine introduction has been confirmed by meta-analyses of these impact studies.^1^^,^^5^

Adenovirus 40/41 was one of the leading causes of severe diarrhea in this study population. This is consistent with results for adenovirus from the GEMS reanalysis^19^ by TAC assay in Kolkata, India, though it was much lower in other sites in Asia and Africa. The two subtypes of adenovirus type F detected in this study (40 and 41) are thought to cause mild diarrhea. Therefore, the high etiological fraction of adenovirus in severe GE cases in children in this analysis and in others with study sites in India^19^^,^^22^ is unexpected, and this pathogen should receive more focused attention as a leading cause of morbidity in children <2 years of age.

*Shigella* spp. was the leading bacterial etiology of severe GE found in this study. This is well corroborated by multiple etiology studies that point to this pathogen as a significant cause of GE in children.^2^^,^^15^^,^^20^^,^^21^

Meta-analyses of norovirus prevalence in GE cases from developing countries have shown that the genogroup NoV GII accounted for more than 90% of norovirus cases.^23^^,^^24^ The AF for norovirus GII in this study was comparable to that of the ROTAVAC efficacy reanalysis in India.^15^ In the GEMS and MAL-ED (Malnutrition and Enteric Disease Study) reanalysis reports,^19^^,^^25^ norovirus GII was among the top six causes of diarrhea in children.

*Cryptosporidium* spp. was the only protozoan among the leading etiologies for severe GE. The adjusted AF for *Cryptosporidium* in Kolkata, India, reported by the GEMS reanalysis data, matched the estimate in this analysis; however, in a similar reanalysis from Niger, the AI estimated from that population was 10 times higher than that estimated here.

The sum of all pathogen-specific AIs was found to be only 66.4% of the overall AI for severe GE. In contrast, similar reanalyses of samples from rotavirus vaccine efficacy trials from Niger (ROTASIIL)^21^ and India (ROTAVAC)^15^ were able to account for 95.2% and 84.7% of cases, respectively. This discrepancy could be due to several reasons, including that rotavirus diarrhea was estimated by EIA and not PCR, meaning the rotavirus AI may have been underestimated, or that a notable proportion of diarrhea was caused by pathogens that were not included in the TAC assays. The list of pathogens tested by TAC in this reanalysis was designed to cover the entire spectrum of diarrhea-causing bacteria, viruses, and parasites commonly thought to be prevalent in India and South Asia. The PCR targets were chosen based on their consistent association with the pathogenic strains of the diarrheal agent from the published literature. However, a few uncommon diarrheagenic pathogens were not tested, such as *Yersinia* spp., *Listeria monocytogenes*, *Clostridium perfringens*, and *Vibrio parahaemolyticus*. In addition, certain PCR targets may be insufficient to detect bacterial pathogens such as the hemolysin A target for *Vibrio cholerae* (*hlyA*); other targets such as *ompW* and *ctxA* may estimate its prevalence more accurately. Regular and periodic diarrheal surveillance of communities in LMICs is essential for detecting novel and emerging agents causing GE in this age group.

When the AI estimates were stratified by age of the child, the pattern of infections at each stage was apparent (Figure 3). The maximum infection rate per the estimated AIs was from 6 to 18 months. This pattern of diarrhea with age is consistent with the reanalysis from Niger, and the reason could be that children who are freshly weaned are more susceptible to gastrointestinal pathogens, and the burden would gradually decrease over the next 12 months as the child develops immunity to the pathogen after infection. This would have implications for vaccine delivery strategy. For example, current efforts focus on developing a *Shigella* vaccine to be administered at 1 year of age, and this would not protect against shigellosis in infants.^26^

Severe GE cases associated with rotavirus tend to be associated with a higher Vesikari score and with higher odds of hospitalization. This is comparable with the profile of rotavirus infections in this age group observed in other studies, in which rotavirus infections in children led to very severe diarrhea in 66.7% of children in a birth cohort^27^ and a study of hospitalized children in India revealed that rotavirus infections accounted for 36.3% of hospital admissions due to diarrhea in the under 5-year age group.^7^

Findings for the clinical profiles of *Shigella* spp., adenovirus, ST-ETEC, and *Cryptosporidium* spp. have parallels in similar analyses in other studies. The ROTAVAC trial reanalysis observed a similar clinical picture for *Cryptosporidium* spp. (increased hospitalizations), *Shigella* spp. (longer duration of diarrhea), and adenovirus (association with vomiting). A similar clinical profile for *Cryptosporidium* and *Shigella* was also observed in the reanalysis of samples from the ROTASIIL efficacy study in Niger.^21^

The adjusted VE estimates do not illustrate a statistically significant difference of outcome in efficacy for the vaccine between the mixed infections and infections by rotavirus alone. Other studies have attempted to do the same for ROTARIX^28^^,^^29^ and the bovine attenuated rotavirus vaccine (RIT 4237)^30^; however, although the efficacy difference in all these studies ranged from 8.0% to 14.0%, none of these effects were statistically significant, which may be attributable to inadequate sample size.

It should also be noted that the estimated AI of rotavirus for severe GE does not appear to be significantly reduced in the vaccinated group as opposed to the placebo group, as the CIs overlap even though the point estimate in the vaccinated group is lower. Again, this is to be expected because the incidence estimates would incorporate the variance from the attribution of etiology and would provide wider intervals as a result, whereas the VE estimates in the parent study would not.

The results from this analysis failed to demonstrate a significant correlation between co-infections with other pathogens causing severe GE and the efficacy of oral rotavirus vaccines in LMICs. This is comparable to results from the ROTAVAC trial reanalysis from India that showed a 11.3% point estimate increase in VE when adjusted for co-infections, although this was not statistically significant. A study in Botswana also did not find evidence of an effect of co-infections on rotavirus vaccine effectiveness.^28^ There may be methodological factors that can explain this discrepancy, including misattribution of the etiology of the disease to rotavirus in the vaccinated group in efficacy trials. Co-infections have been hypothesized to interfere with vaccine response through multiple means, such as competition for cell entry and replication or for cellular factors required in transcription, replication, and assembly.^12^^,^^13^ Other biological causes for this lower efficacy could be chronic malnutrition prevalent in children in LMICs, gut inflammation, environmental enteropathy, and interference from antibodies in the system that would lead to a suboptimal immune response. The increased infective load in LMICs due to poor hygienic conditions also could be a factor in the reduced ability of the vaccine to protect against infections when compared with results in high-income settings; a diarrheal state with altered mucosa and rapid gut transit may interfere with re-uptake of the vaccine. Rotavirus replication has been shown to be impaired by enteroviruses,^31^ and a study from Bangladesh demonstrated lowered rotavirus seroconversion on concomitant administration of the monovalent rotavirus vaccine and trivalent oral polio vaccine vaccine.^32^ However, the parent vaccine trial of this study did not find any effect on seroconversion with OPV administration.^10^ This reanalysis included only targets that were known to cause diarrhea in this age group. The presence of commensals such as enteroviruses could also interfere with efficacy, and further studies in samples with those commensals as targets or a metagenomic analysis in vaccinated cohorts between sites could lead to a better understanding of the interactions between the VE of viruses and organisms present in the gut microbiome.

This study attempted to account for endpoint misclassifications for SRVGE cases in the parent phase III vaccine trial that could lead to lower estimates of VE of ROTASIIL in LMIC settings. This misclassification can be expected to vary inversely with the strength of the association of pathogen quantities in the stool and disease and is therefore even more relevant for enteric pathogens such as norovirus GII and *Cryptosporidium* spp. Pathogens such as norovirus GII and *Campylobacter* spp., which are commonly detected from diarrheal stool but less likely to be the cause of diarrhea,^24^^,^^33^ would be especially susceptible to this misclassification in vaccine trials. Approaches similar to that used in this study—testing for other enteric pathogens in phase III vaccine trials of these pathogens—could allow us to assess this.

This study had several limitations. The samples selected for testing from the parent study did not include the non-severe non-rotavirus GE cases. This could lead to a bias in the attribution of pathogens for severe GE that usually cause milder infections or could pass through the gastrointestinal tract as commensals (e.g., adenovirus). Another limitation was that rotavirus positivity was estimated by EIA, which is not as sensitive as a molecular assay, and this could underestimate the attribution of rotavirus to severe GE, which would explain the gap between any-cause AI and the combined AI from all measured targets. The use of the Vesikari score to categorize the severity of diarrhea could bias AFs toward viral etiologies, as vomiting, which is a component in the scoring system, is relatively more frequent in viral GE than with bacterial and protozoan etiologies.

## CONCLUSION

In summary, the quantitative output of the TAC assay is a useful tool to analyze etiologies of disease in mixed samples that could have commensals in addition to multiple pathogenic organisms. The analysis brings to our attention the multiple etiologies of severe GE in children of a very young age, which include adenovirus, *Shigella*, norovirus, *Cryptosporidium*, and ETEC. Future public health and vaccination strategies should be planned based on studies carried out locally to determine the agents of concern. Although the adjusted VE estimate obtained was not sufficient to conclude whether co-infections are indeed the cause of poorer vaccine performance in LMICs, it is likely that this is not a major contributor, and future trials must include analysis of other factors such as the gut microbiome, interactions with other vaccines, gut inflammation, and malnutrition status.

## Supplemental Materials

10.4269/ajtmh.23-0348 Supplemental Materials

## References

[1] Burnett E Parashar UD Tate JE, 2020. Real-world effectiveness of rotavirus vaccines, 2006–19: A literature review and meta-analysis. Lancet Glob Health 8: e1195–e1202. 32827481 10.1016/S2214-109X(20)30262-X PMC8097518

[2] Platts-Mills JA , 2017. Impact of rotavirus vaccine introduction and postintroduction etiology of diarrhea requiring hospital admission in Haydom, Tanzania, a rural African setting. Clin Infect Dis 65: 1144–1151. 28575304 10.1093/cid/cix494 PMC5850044

[3] Patel MM Steele D Gentsch JR Wecker J Glass RI Parashar UD, 2011. Real-world impact of rotavirus vaccination. Pediatr Infect Dis J 30: S1–S5. 21183833 10.1097/INF.0b013e3181fefa1f

[4] Schwartz LM Halloran ME Rowhani-Rahbar A Neuzil KM Victor JC, 2017. Rotavirus vaccine effectiveness in low-income settings: An evaluation of the test-negative design. Vaccine 35: 184–190. 27876198 10.1016/j.vaccine.2016.10.077 PMC5154240

[5] Velázquez RF Linhares AC Muñoz S Seron P Lorca P DeAntonio R Ortega-Barria E, 2017. Efficacy, safety and effectiveness of licensed rotavirus vaccines: A systematic review and meta-analysis for Latin America and the Caribbean. BMC Pediatr 17: 14. 28086819 10.1186/s12887-016-0771-y PMC5237165

[6] Becker-Dreps S , 2014. Etiology of childhood diarrhea after rotavirus vaccine introduction: A prospective, population-based study in Nicaragua. Pediatr Infect Dis J 33: 1156–1163. 24879131 10.1097/INF.0000000000000427 PMC4216626

[7] Varghese T , 2021. Rotavirus strain distribution before and after introducing rotavirus vaccine in India. Pathogens 10: 416. 33915946 10.3390/pathogens10040416 PMC8066972

[8] Clark A van Zandvoort K Flasche S Sanderson C Bines J Tate J Parashar U Jit M, 2019. Efficacy of live oral rotavirus vaccines by duration of follow-up: A meta-regression of randomised controlled trials. Lancet Infect Dis 19: 717–727. 31178289 10.1016/S1473-3099(19)30126-4 PMC6595176

[9] Bhandari N , 2014. Efficacy of a monovalent human-bovine (116E) rotavirus vaccine in Indian infants: A randomised double blind placebo controlled trial. Lancet 383: 2136–2143. 24629994 10.1016/S0140-6736(13)62630-6 PMC4532697

[10] Kulkarni PS , 2017. A randomized phase III clinical trial to assess the efficacy of a bovine-human reassortant pentavalent rotavirus vaccine in Indian infants. Vaccine 35: 6228–6237. 28967523 10.1016/j.vaccine.2017.09.014 PMC5651219

[11] Kvistgaard AS Pallesen LT Arias CF López S Petersen TE Heegaard CW Rasmussen JT, 2004. Inhibitory effects of human and novine milk constituents on rotavirus infections. J Dairy Sci 87: 4088–4096. 15545370 10.3168/jds.S0022-0302(04)73551-1

[12] Parker EP Ramani S Lopman BA Church JA Iturriza-Gómara M Prendergast AJ Grassly NC, 2018. Causes of impaired oral vaccine efficacy in developing countries. Future Microbiol 13: 97–118. 29218997 10.2217/fmb-2017-0128 PMC7026772

[13] Patriarca PA Wright PF John TJ, 1991. Factors affecting the immunogenicity of oral poliovirus vaccine in developing countries: Review. Rev Infect Dis 13: 926–939. 1660184 10.1093/clinids/13.5.926

[14] Taniuchi M , 2016. Impact of enterovirus and other enteric pathogens on oral polio and rotavirus vaccine performance in Bangladeshi infants. Vaccine 34: 3068–3075. 27154394 10.1016/j.vaccine.2016.04.080 PMC4912219

[15] Praharaj I , 2019. Diarrheal etiology and impact of coinfections on rotavirus vaccine efficacy estimates in a clinical trial of a monovalent human–bovine (116E) oral rotavirus vaccine, Rotavac, India. Clin Infect Dis 69: 243–250. 30335135 10.1093/cid/ciy896 PMC6603264

[16] Ruuska T Vesikari T, 1990. Rotavirus disease in Finnish children: Use of numerical scores for clinical severity of diarrhoeal episodes. Scand J Infect Dis 22: 259–267. 2371542 10.3109/00365549009027046

[17] Liu J , 2016. Optimization of quantitative PCR methods for enteropathogen detection. PLoS One 11: e0158199. 27336160 10.1371/journal.pone.0158199 PMC4918952

[18] Bruzzi P Green SB Byar DP Brinton LA Schairer C, 1985. Estimating the population attributable risk for multiple risk factors using case-control data. Am J Epidemiol 122: 904–914. 4050778 10.1093/oxfordjournals.aje.a114174

[19] Liu J , 2016. Use of quantitative molecular diagnostic methods to identify causes of diarrhoea in children: A reanalysis of the GEMS case-control study. Lancet 388: 1291–1301. 27673470 10.1016/S0140-6736(16)31529-X PMC5471845

[20] Lewnard JA Rogawski McQuade ET Platts-Mills JA Kotloff KL Laxminarayan R, 2020. Incidence and etiology of clinically-attended, antibiotic-treated diarrhea among children under five years of age in low- and middle-income countries: Evidence from the Global Enteric Multicenter Study. PLoS Negl Trop Dis 14: e0008520. 32776938 10.1371/journal.pntd.0008520 PMC7444547

[21] Platts-Mills JA , 2021. Etiology and incidence of moderate-to-severe diarrhea in young children in Niger. J Pediatric Infect Dis Soc 10: 1062–1070. 34468743 10.1093/jpids/piab080 PMC8719619

[22] Chandra P , 2021. Genetic characterization and phylogenetic variations of human adenovirus‐F strains circulating in eastern India during 2017–2020. J Med Virol 93: 6180–6190. 34138479 10.1002/jmv.27136

[23] Nguyen GT Phan K Teng I Pu J Watanabe T, 2017. A systematic review and meta-analysis of the prevalence of norovirus in cases of gastroenteritis in developing countries. Medicine (Baltimore) 96: e8139. 28984764 10.1097/MD.0000000000008139 PMC5738000

[24] Mans J, 2019. Norovirus infections and disease in lower‐middle and low‐income countries, 1997–2018. Viruses 11: 341. 30974898 10.3390/v11040341 PMC6521228

[25] Platts-Mills JA , 2018. Use of quantitative molecular diagnostic methods to assess the aetiology, burden, and clinical characteristics of diarrhoea in children in low-resource settings: A reanalysis of the MAL-ED cohort study. Lancet Glob Health 6: e1309–e1318. 30287127 10.1016/S2214-109X(18)30349-8 PMC6227251

[26] WHO , 2021. WHO Preferred Product Characteristics for Vaccines against Shigella. Geneva, Switzerland: World Health Organization.

[27] Paul A Gladstone BP Mukhopadhya I Kang G, 2014. Rotavirus infections in a community based cohort in Vellore, India. Vaccine 32: A49–A54. 25091680 10.1016/j.vaccine.2014.03.039

[28] Mokomane M Tate JE Steenhoff AP Esona MD Bowen MD Lechiile K Pernica JM Kasvosve I Parashar UD Goldfarb DM, 2018. Evaluation of the influence of gastrointestinal coinfections on rotavirus vaccine effectiveness in Botswana. Pediatr Infect Dis J 37: e58–e62. 29189612 10.1097/INF.0000000000001828 PMC5807168

[29] Groome MJ , 2014. Effectiveness of monovalent human rotavirus vaccine against admission to hospital for acute rotavirus diarrhoea in South African children: A case-control study. Lancet Infect Dis 14: 1096–1104. 25303843 10.1016/S1473-3099(14)70940-5

[30] Lanata CF Black RE del Aguila R Gil A Verastegui H Gerna G Flores J Kapikian Albert Z Andre FE, 1989. Protection of Peruvian children against rotavirus diarrhea of specific serotypes by one, two, or three doses of the RIT 4237 attenuated bovine rotavirus vaccine. J Infect Dis 159: 452–459. 2536789 10.1093/infdis/159.3.452

[31] Wang H Moon S Wang Y Jiang B, 2012. Multiple virus infection alters rotavirus replication and expression of cytokines and Toll-like receptors in intestinal epithelial cells. Virus Res 167: 48–55. 22497974 10.1016/j.virusres.2012.04.001

[32] Emperador DM Velasquez DE Estivariz CF Lopman B Jiang B Parashar U Anand A Zaman K, 2016. Interference of monovalent, bivalent, and trivalent oral poliovirus vaccines on monovalent rotavirus vaccine immunogenicity in rural Bangladesh. Clin Infect Dis 62: 150–156. 26349548 10.1093/cid/civ807 PMC4755336

[33] Platts-Mills JA , 2014. Detection of *Campylobacter* in stool and determination of significance by culture, enzyme immunoassay, and PCR in developing countries. J Clin Microbiol 52: 1074–1080. 24452175 10.1128/JCM.02935-13 PMC3993515

