# Supplementary material for: Effect of Non-Rotavirus Enteric Infections on Vaccine Efficacy in a ROTASIIL Clinical Trial
Source: Am J Trop Med Hyg. 2024 Apr 16;110(6):1201–9. doi: 10.4269/ajtmh.23-0348 (PMC11154053; doi:10.4269/ajtmh.23-0348)
Supplement: Supplemental Materials [file tpmd230348.SD1.pdf]

Supplementary table 1: Targets for the Taqman Array Cards used to detect pathogens causing diarrhea/gastroenteritis

**Pathogen**	**Gene Target**
Adenovirus 40/41	Fiber gene[1]
Adenovirus (pan)	Hexon[2]
Astrovirus	Capsid[3]
Norovirus GI	ORF 1-2[3]
Norovirus GII	ORF 1-2[3]
Sapovirus	RdRp[2]
EAEC*	*aaic*[2]
	aatA[2]
tEPEC**	*eae*[2]
	bfpa[2]
ETEC†	LT[2]
	STh[2]
	STp[2]
STEC††	*stx1/stx2*[3]
Aeromonas	Aerolysin[3]
Bacteroides fragilis	EGBF
C.jejuni/C.coli	*cadF*[1]
Campylobacter spp	Cpn60[1]
Clostridium difficile	*tcdB & tcdA*[3]
Helicobacter pylori	*ureC*[4]
Salmonella enterica	*ttr*[1]
Shigella/EIEC§	*ipaH*[3]
Vibrio cholerae	*hlyA*[2]
Encephalitozoan intestinalis	SSU rRNA[1]
Enterozytozoon bieneusi	ITS[1]
Cryptosporidium spp.	18S rRNA[3]
Cryptosporidium hominis/parvum	*LIB13*[1]
Giardia lamblia	18S rRNA[1]
Cyclospora cayetonesis	18S rRNA[1]
Cystoisopora belli	18S rRNA[1]

*enteroaggregative *Escherichia coli;* **typical enteropathogenic *Escherichia coli;* †enterotoxigenic *Escherichia coli;* ††Shiga-Toxin-Producing *Escherichia coli;* § enteroinvasive *Escherichia coli*

References

1. Liu J, Gratz J, Amour C, Nshama R, Walongo T, Maro A, Mduma E, Platts-Mills J, Boisen N, Nataro J, Haverstick DM, Kabir F, Lertsethtakarn P, Silapong S, Jeamwattanalert P, et al., 2016. Optimization of Quantitative PCR Methods for Enteropathogen Detection. PLoS ONE 11: e0158199

2. Liu J, Kabir F, Manneh J, Lertsethtakarn P, Begum S, Gratz J, Becker SM, Operario DJ, Taniuchi M, Janaki L, Platts-Mills JA, Haverstick DM, Kabir M, Sobuz SU, Nakjarung K, et al., 2014. Development and assessment of molecular diagnostic tests for 15 enteropathogens causing childhood diarrhoea: a multicentre study. The Lancet Infectious Diseases 14: 716–724

3. Liu J, Gratz J, Amour C, Kibiki G, Becker S, Janaki L, Verweij JJ, Taniuchi M, Sobuz SU, Haque R, Haverstick DM, Houpt ER., 2013. A laboratory-developed TaqMan Array Card for simultaneous detection of 19 enteropathogens. J Clin Microbiol 51: 472–480

4. Liu J, Platts-Mills JA, Juma J, Kabir F, Nkeze J, Okoi C, Operario DJ, Uddin J, Ahmed S, Alonso PL, Antonio M, Becker SM, Blackwelder WC, Breiman RF, Faruque ASG, et al., 2016. Use of quantitative molecular diagnostic methods to identify causes of diarrhoea in children: a reanalysis of the GEMS case-control study. The Lancet 388: 1291–1301

Supplementary table 2: Number of samples chosen to be tested in each group from the parent vaccine efficacy trial

Type of sample	BRV-PV	Placebo	Total
Severe RVGE* case	190	304	494
Non-severe RVGE case	373	408	781
Severe non-RVGE case	744	675	1,419
Total	1,307	1,387	2,694

*Rotavirus gastroenteritis

Supplementary table 3: Baseline clinical characteristics of samples from the diarrheal episodes selected for analysis

Characteristics	Categories	Frequency (%)
Study Group	BRV-PV* group	1,284 (48·5%)
	Placebo group	1,364 (51·5%)
RVGE**	No	1,373 (51·9%)
	Yes	1,275 (48·1%)
SRVGE***	No	2,154 (81·3%)
	Yes	494 (18·7%)
Sex	Female	1,236 (46·7%)
	Male	1,412 (53·3%)
Duration of Diarrhea	<5	1,582 (59·7%)
	≥5	1,066 (40·3%)
Dehydration	1-5%	632 (89·8%)
	≥6%	72 (10·2%)
Hospitalized	No	1,938 (73·2%)
	Yes	710 (26·8%)
Vesikari	<15	2,230 (84·2%)
	≥15	418 (15·8%)
Maximum number of vomiting	0	475 (17·9%)
	1	323 (12·2%)
	2-4	1,389 (52·5%)
	≥5	461 (17·4%)
Age (days)		Min: 42, Mean – 364.3, Max:734

*Bovine Rotavirus – Pentavalent Vaccine; **rotavirus gastroenteritis; ***severe rotavirus gastroenteritis

Supplementary table 4. Attributable incidence of all-cause and etiology-specific severe diarrhea, per 1000 child-years

	Any cause	Rotavirus	Adenovirus 40/41	*Shigella*	Norovirus GII	ST-ETEC*	*Cryptosporidium*	Sapovirus	Astrovirus	*C. jejuni/coli*	*V. cholerae*
**Overall**	131.7	31.0 (20.1, 33.5)	22.4 (17.2, 27.1)	10.5 (7.9, 15.8)	5.2 (0.0, 11.1)	5.0 (3.0, 6.4)	4.3 (2.9, 5.4)	2.9 (0.0, 11.8)	2.9 (0.0, 5.1)	2.4 (0.0, 5.4)	0.8 (0.4, 1.0)
Age											
**0-5 months**	102.9	9.9 (6.2, 10.7)	15.3 (11.6, 18.6)	1.6 (1.2, 2.7)	2.1 (0.0, 4.8)	1.8 (1.1, 2.4)	0.2 (0.1, 0.2)	1.3 (0.0, 4.6)	1.2 (0.0, 2.2)	1.0 (0.0, 2.4)	0.3 (0.1, 0.3)
**6-11 months**	187.5	45.3 (29.0, 49.0)	32.6 (24.7, 38.9)	11.7 (8.7, 17.8)	9.0 (0.0, 19.0)	6.7 (4.2, 8.7)	8.4 (5.5, 10.5)	4.0 (0.0, 16.6)	4.2 (0.0, 7.4)	4.5 (0.0, 9.9)	0.6 (0.3, 0.8)
**12-17 months**	151.1	47.9 (31.1, 51.5)	24.3 (18.3, 29.2)	11.2 (8.5, 17.6)	6.2 (0.0, 13.8)	5.4 (3.1, 7.1)	4.4 (2.9, 5.6)	3.5 (0.0, 14.3)	3.2 (0.0, 5.9)	2.5 (0.0, 5.8)	1.2 (0.6, 1.5)
**18-23 months**	79.2	16.4 (10.3, 17.8)	16.1 (12.3, 19.6)	15.5 (12.1, 22.5)	2.4 (0.0, 5.4)	5.4 (3.3, 7.0)	3.3 (2.3, 4.2)	2.4 (0.0, 9.1)	2.6 (0.0, 4.6)	1.4 (0.0, 3.2)	1.0 (0.5, 1.1)
Hospitalization											
**Yes**	48.5	15.9 (10.3, 17.2)	8.2 (6.2, 9.9)	2.7 (2.1, 4.1)	1.8 (0.0, 4.1)	1.3 (0.8, 1.7)	1.6 (1.1, 2.0)	1.0 (0.0, 3.9)	1.2 (0.0, 2.1)	0.7 (0.0, 1.5)	0.4 (0.2, 0.4)
**No**	83.2	15.1 (9.9, 16.3)	14.3 (10.6, 17.3)	7.7 (5.9, 11.8)	3.3 (0.0, 7.2)	3.7 (2.1, 4.8)	2.7 (1.8, 3.4)	1.7 (0.0, 8.1)	1.7 (0.0, 3.1)	1.8 (0.0, 4.0)	0.4 (0.2, 0.5)
Received BRV-PV											
**Overall**	128.5	24.0 (15.3, 25.9)	23.9 (17.5, 28.9)	10.8 (8.3, 16.1)	5.4 (0.0, 11.8)	5.7 (3.2, 7.5)	4.4 (3.0, 5.5)	2.6 (0.0, 12.1)	2.6 (0.0, 4.6)	2.4 (0.0, 5.3)	0.4 (0.1, 0.6)
**< 12 months**	145.9	23.7 (15.2, 25.5)	25.8 (19.7, 31.2)	6.9 (5.3, 10.5)	6.2 (0.0, 13.8)	5.3 (3.2, 6.8)	4.5 (3.1, 5.6)	2.8 (0.0, 11.6)	2.3 (0.0, 4.2)	3.1 (0.0, 6.6)	0.2 (0.0, 0.3)
**12-23 months**	113.3	24.3 (16.0, 26.2)	22.0 (16.2, 26.6)	14.4 (11.2, 21.1)	4.7 (0.0, 9.9)	6.2 (3.8, 8.1)	4.2 (2.9, 5.4)	2.8 (0.0, 12.0)	2.8 (0.0, 5.0)	2.0 (0.0, 4.2)	0.6 (0.2, 0.8)
Received placebo											
**Overall**	134.9	38.2 (24.5, 41.1)	21.1 (16.2, 25.8)	10.0 (7.7, 15.5)	5.1 (0.0, 10.8)	4.2 (2.5, 5.5)	4.2 (2.9, 5.4)	3.1 (0.0, 12.0)	3.2 (0.0, 5.6)	2.5 (0.0, 5.5)	1.2 (0.6, 1.4)
**< 12 months**	155.8	36.2 (23.5, 39.2)	24.5 (18.5, 29.2)	7.6 (5.7, 12.0)	5.6 (0.0, 12.4)	3.9 (2.2, 5.0)	5.2 (3.7, 6.5)	3.0 (0.0, 11.9)	3.6 (0.0, 6.1)	3.1 (0.0, 6.6)	0.7 (0.3, 0.9)
**12-23 months**	116.5	39.6 (24.6, 42.8)	18.4 (13.9, 22.2)	12.1 (9.4, 18.6)	4.0 (0.0, 9.4)	4.5 (2.7, 6.0)	3.4 (2.3, 4.4)	3.0 (0.0, 11.5)	2.9 (0.0, 5.4)	1.9 (0.0, 4.3)	1.6 (0.8, 1.8)

* heat-stable toxin-producing enterotoxigenic *Escherichia coli*
